# (*E*)-1-(2,4-Dinitro­phen­yl)-2-[1-(3-meth­oxy­phen­yl)ethyl­idene]hydrazine

**DOI:** 10.1107/S1600536812026979

**Published:** 2012-06-20

**Authors:** Hoong-Kun Fun, Suchada Chantrapromma, Boonlerd Nilwanna, Thawanrat Kobkeatthawin

**Affiliations:** a X-ray Crystallography Unit, School of Physics, Universiti Sains Malaysia, 11800 USM, Penang, Malaysia; b Crystal Materials Research Unit, Department of Chemistry, Faculty of Science, Prince of Songkla University, Hat-Yai, Songkhla 90112, Thailand

## Abstract

There are two crystallographically independent mol­ecules in the asymmetric unit of the title compound, C_15_H_14_N_4_O_5_, with different conformations for the meth­oxy groups. The mol­ecules are both slightly twisted, the dihedral angles between two benzene rings being 8.37 (18)° in one and 7.31 (18)° in the other. In both mol­ecules, the two nitro groups are essentially coplanar with their bound benzene ring, with the r.m.s. deviation of the dinitro­benzene plane being 0.0310 (3) Å in one mol­ecule and 0.0650 (3) Å in the other. In each mol­ecule, an intra­molecular N—H⋯O hydrogen bond generates an *S*(6) ring motif. In the crystal, mol­ecules are linked by weak C—H⋯O inter­actions and stacked along the *a* axis through π–π inter­actions, with centroid–centroid distances of 3.651 (2) and 3.721 (2) Å. The crystal studied was a non-merohedral twin with a refined minor component of 20.1 (3)%.

## Related literature

For bond-length data, see: Allen *et al.* (1987 ▶). For hydrogen-bond motifs, see: Bernstein *et al.* (1995 ▶). For related structures, see: Chantrapromma *et al.* (2012 ▶); Fun *et al.* (2010 ▶); Nilwanna *et al.* (2011 ▶). For background to the biological activity of hydro­zones, see: Bendre *et al.* (1998 ▶); El-Sherif (2009 ▶); Gokce *et al.* (2009 ▶); Molyneux (2004 ▶); Sathyadevi *et al.* (2012 ▶); Xia *et al.* (2008 ▶).
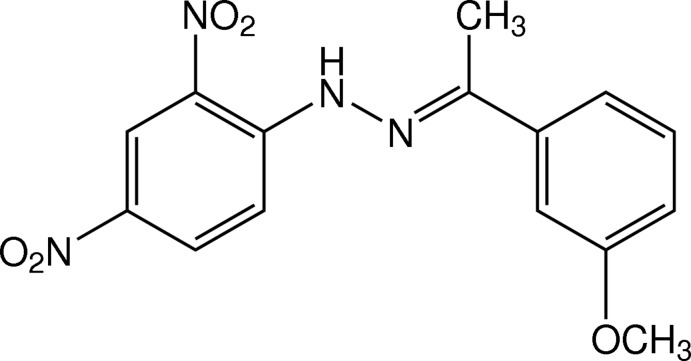


## Experimental

### 

#### Crystal data

C_15_H_14_N_4_O_5_
*M* *_r_* = 330.30
Triclinic, 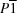

*a* = 7.5612 (13) Å
*b* = 10.4517 (18) Å
*c* = 19.516 (3) Å
α = 76.034 (4)°
β = 89.531 (4)°
γ = 84.052 (4)°
*V* = 1488.4 (4) Å^3^
*Z* = 4
Mo *K*α radiation
μ = 0.11 mm^−1^
*T* = 100 K
0.33 × 0.14 × 0.05 mm

#### Data collection

Bruker SMART APEXII DUO CCD area-detector diffractometer
Absorption correction: multi-scan (*SADABS*; Bruker, 2009 ▶) *T*_min_ = 0.964, *T*_max_ = 0.994
7846 measured reflections
7846 independent reflections
5592 reflections with *I* > 2σ(*I*)

#### Refinement

*R*[*F* ^2^ > 2σ(*F* ^2^)] = 0.086
*wR*(*F* ^2^) = 0.296
*S* = 1.11
7846 reflections
444 parameters
2 restraints
H atoms treated by a mixture of independent and constrained refinement
Δρ_max_ = 0.53 e Å^−3^
Δρ_min_ = −0.59 e Å^−3^

### 

Data collection: *APEX2* (Bruker, 2009 ▶); cell refinement: *SAINT* (Bruker, 2009 ▶); data reduction: *SAINT*; program(s) used to solve structure: *SHELXTL* (Sheldrick, 2008 ▶); program(s) used to refine structure: *SHELXTL*; molecular graphics: *SHELXTL*; software used to prepare material for publication: *SHELXTL* and *PLATON* (Spek, 2009 ▶).

## Supplementary Material

Crystal structure: contains datablock(s) global, I. DOI: 10.1107/S1600536812026979/is5147sup1.cif

Structure factors: contains datablock(s) I. DOI: 10.1107/S1600536812026979/is5147Isup2.hkl

Supplementary material file. DOI: 10.1107/S1600536812026979/is5147Isup3.cml

Additional supplementary materials:  crystallographic information; 3D view; checkCIF report

## Figures and Tables

**Table 1:** Hydrogen-bond geometry (Å, °)

*D*—H⋯*A*	*D*—H	H⋯*A*	*D*⋯*A*	*D*—H⋯*A*
N1*A*—H1*NA*⋯O1*A*	0.87 (3)	1.94 (4)	2.611 (4)	133 (3)
N1*B*—H1*NB*⋯O1*B*	0.88 (4)	1.96 (3)	2.611 (4)	130 (4)
C5*B*—H5*B*⋯O3*A* ^i^	0.93	2.59	3.462 (5)	156
C6*B*—H6*B*⋯O4*A* ^i^	0.93	2.50	3.277 (4)	141
C8*A*—H8*C*⋯O3*B* ^ii^	0.96	2.57	3.376 (5)	142
C8*B*—H8*E*⋯O4*B* ^iii^	0.96	2.45	3.402 (5)	170
C12*B*—H12*B*⋯O2*A* ^iv^	0.93	2.57	3.447 (5)	157
C13*A*—H13*A*⋯O3*B* ^iii^	0.93	2.48	3.204 (5)	135
C13*B*—H13*B*⋯O3*A* ^iv^	0.93	2.55	3.448 (5)	162
C14*B*—H14*B*⋯O4*B* ^iii^	0.93	2.47	3.338 (5)	155
C15*B*—H15*E*⋯O3*A* ^v^	0.96	2.57	3.301 (5)	133

Symmetry codes: (i) 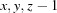; (ii) 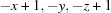; (iii) 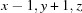; (iv) 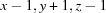; (v) 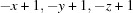.

**Fig. 1.**
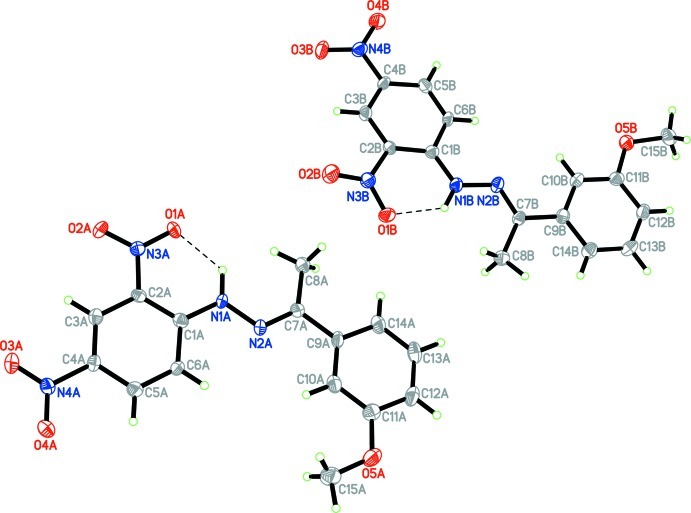
The molecular structure of the title compound, showing 60% probability displacement ellipsoids and the atom-numbering scheme. Intramolecular N—H···O hydrogen bonds are shown as dashed lines.

**Fig. 2.**
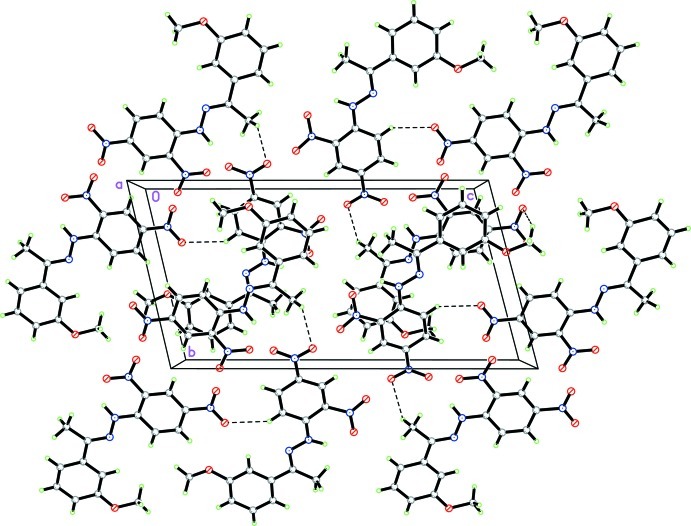
The crystal packing of the title compound, viewed approximately along the a axis. Hydrogen bonds are shown as dashed lines.

## supplementary crystallographic information

### Comment

Hydrazones are important compounds which have considerable interesting applications involving biological activities such as antibacterial (El-Sherif *et al.*, 2009), antioxidant (Sathyadevi *et al.*, 2012), anticancer (Xia *et al.*, 2008), anti-inflammatory (Gokce *et al.*, 2009) and tyrosinase inhibitory (Bendre *et al.*, 1998) activities. With our on-going research on crystal structures, bioactivity and antioxidant activity of hydrazones (Chantrapromma *et al.*, 2012; Fun *et al.*, 2010; Nilwanna *et al.*, 2011), the title compound (I) was synthesized. The evaluation of its antioxidant activity by DPPH scavenging (Molyneux, 2004) was found to be inactive. Herein we report the synthesis and crystal structure of (I).

In Fig. 1, there are two crystallographically independent molecules *A* and *B* in the asymmetric unit of (I), C_15_H_14_N_4_O_5_, with differences in bond angles and conformations of the methoxy groups in which in molecule *A* the methoxy group is co-planar with its bound benzene ring and pointed toward the central ethylidenehydrazine (N1/N2/C7/C8) as indicated by the torsion angle C15A–O5A–C11A–C10A = -2.6 (5)°, whereas in molecule *B* it is twisted and pointed away from the central ethylidenehydrazine with the torsion angle C15B–O5B–C11B–C10B = 167.7 (3)°. The molecular structure of (I) is twisted with the dihedral angle between the two benzene rings being 8.37 (18)° in molecule *A* and 7.31 (18)° in molecule *B*. The central ethylidenehydrazine bridge is planar with the torsion angles N1–N2–C7–C8 = -1.3 (5) and 1.7 (5)° in molecules *A* and *B*, respectively. The mean plane through this central bridge makes dihedral angles of 9.0 (2) and 1.5 (2)° with the 2,4-dinitro- and 3-methoxy-substituted benzene rings, respectively, in molecule *A*, whereas the corresponding values are 7.8 (2) and 1.0 (2)° in molecule *B*. In both molecules, the two nitro groups are co-planar with their bound benzene rings with *r.m.s*. deviations of 0.0310 (3) and 0.0650 (3) Å in molecules *A* and *B*, respectively, for the twelve non H-atoms (C1–C6/N3/N4/O1–O4). In each molecule, intramolecular N—H···O hydrogen bonds (Fig. 1 and Table 1) generate two S(6) ring motifs (Bernstein *et al.*, 1995). The bond distances are in normal ranges (Allen *et al.*, 1987) and are comparable with the related structures (Chantrapromma *et al.*, 2012; Fun *et al.*, 2010; Nilwanna *et al.*, 2011).

In the crystal packing (Fig. 2), the molecules are linked by weak C—H···O interactions (Table 1) and stacked along the *a* axis by π–π interactions with distances of *Cg*1···*Cg*4^v^ = 3.721 (2) Å and *Cg*2···*Cg*3^v^ = 3.651 (2) Å; *Cg*1, *Cg*2, *Cg*3 and *Cg*4 are the centroids of C1A–C6A, C9A–C14A, C1B–C6B and C9B–C14B benzene rings, respectively.

### Experimental

The title compound (I) was synthesized by dissolving 2,4-dinitrophenylhydrazine (0.40 g, 2 mmol) in ethanol (10.00 ml) and H_2_SO_4_ (conc.) (98 %, 0.50 ml) was slowly added with stirring. 3-Methoxyacetophenone (0.33 ml, 2 mmol) was then added to the solution with continuous stirring. The solution was stirred for 1 hr yielding an orange solid, which was filtered off and washed with methanol. Orange plate-shaped single crystals of the title compound suitable for *X*-ray structure determination were recrystallized from ethanol by slow evaporation of the solvent at room temperature over several days (m.p. 459-460 K).

### Refinement

Amide H atoms were located in a difference Fourier map and were refined with a distance restraint of N—H = 0.86 (2) Å and with *U*_iso_(H) = 1.5*U*_eq_(N). The remaining H atoms were positioned geometrically and allowed to ride on their parent atoms, with C—H = 0.93 Å for aromatic and 0.96 Å for CH_3_ atoms. The *U*_iso_ values were constrained to be 1.5*U*_eq_ of the carrier atom for methyl H atoms and 1.2*U*_eq_ for the remaining H atoms. A rotating group model was used for the methyl groups. The crystal studied was a twin with BASF = 0.201 (3). As the twin law is non-integer, PLATON was used to convert the original data set in HKLF4 format into HKLF5 format for the final refinement. This method would reduce all the R-values of the data set to zeros. In the submission CIF, the R-values of the original data set in HKLF4 format was inputted in these fields in place of the zeros.

### Crystal data

**Table:**

C_15_H_14_N_4_O_5_	*Z* = 4
*M**_r_* = 330.30	*F*(000) = 688
Triclinic, *P*1	*D*_x_ = 1.474 Mg m^−^^3^
Hall symbol: -P 1	Mo *K*α radiation, λ = 0.71073 Å
*a* = 7.5612 (13) Å	Cell parameters from 7846 reflections
*b* = 10.4517 (18) Å	θ = 1.1–29.0°
*c* = 19.516 (3) Å	µ = 0.11 mm^−^^1^
α = 76.034 (4)°	*T* = 100 K
β = 89.531 (4)°	Plate, orange
γ = 84.052 (4)°	0.33 × 0.14 × 0.05 mm
*V* = 1488.4 (4) Å^3^	

### Data collection

**Table:**

Bruker SMART APEXII DUO CCD area-detector diffractometer	7846 independent reflections
Radiation source: fine-focus sealed tube	5592 reflections with *I* > 2σ(*I*)
Graphite monochromator	*R*_int_ = 0.000
φ and ω scans	θ_max_ = 29.0°, θ_min_ = 1.1°
Absorption correction: multi-scan (*SADABS*; Bruker, 2009)	*h* = −10→10
*T*_min_ = 0.964, *T*_max_ = 0.994	*k* = −13→14
7846 measured reflections	*l* = −12→26

### Refinement

**Table:**

Refinement on *F*^2^	Primary atom site location: structure-invariant direct methods
Least-squares matrix: full	Secondary atom site location: difference Fourier map
*R*[*F*^2^ > 2σ(*F*^2^)] = 0.086	Hydrogen site location: inferred from neighbouring sites
*wR*(*F*^2^) = 0.296	H atoms treated by a mixture of independent and constrained refinement
*S* = 1.11	*w* = 1/[σ^2^(*F*_o_^2^) + (0.1531*P*)^2^ + 2.363*P*] where *P* = (*F*_o_^2^ + 2*F*_c_^2^)/3
7846 reflections	(Δ/σ)_max_ = 0.001
444 parameters	Δρ_max_ = 0.53 e Å^−^^3^
2 restraints	Δρ_min_ = −0.59 e Å^−^^3^

### Special details

**Table:**

Experimental. The crystal was placed in the cold stream of an Oxford Cryosystems Cobra open-flow nitrogen cryostat (Cosier & Glazer, 1986) operating at 100.0 (1) K.
Geometry. All esds (except the esd in the dihedral angle between two l.s. planes) are estimated using the full covariance matrix. The cell esds are taken into account individually in the estimation of esds in distances, angles and torsion angles; correlations between esds in cell parameters are only used when they are defined by crystal symmetry. An approximate (isotropic) treatment of cell esds is used for estimating esds involving l.s. planes.
Refinement. Refinement of F^2^ against ALL reflections. The weighted R-factor wR and goodness of fit S are based on F^2^, conventional R-factors R are based on F, with F set to zero for negative F^2^. The threshold expression of F^2^ > 2sigma(F^2^) is used only for calculating R-factors(gt) etc. and is not relevant to the choice of reflections for refinement. R-factors based on F^2^ are statistically about twice as large as those based on F, and R- factors based on ALL data will be even larger.

### Fractional atomic coordinates and isotropic or equivalent isotropic displacement parameters (Å^2^)

**Table:**

	*x*	*y*	*z*	*U*_iso_*/*U*_eq_	
O1A	0.5983 (4)	0.0757 (3)	0.79029 (14)	0.0199 (6)	
O2A	0.7040 (4)	−0.0325 (3)	0.89291 (14)	0.0181 (6)	
O3A	0.6439 (4)	0.1398 (3)	1.09429 (14)	0.0196 (6)	
O4A	0.4793 (4)	0.3241 (3)	1.09095 (15)	0.0233 (6)	
O5A	−0.0748 (4)	0.8559 (3)	0.67880 (16)	0.0231 (6)	
N1A	0.4046 (4)	0.3030 (3)	0.77458 (15)	0.0128 (6)	
H1NA	0.442 (6)	0.238 (3)	0.756 (2)	0.019*	
N2A	0.3056 (4)	0.4176 (3)	0.73966 (16)	0.0146 (6)	
N3A	0.6176 (4)	0.0642 (3)	0.85475 (16)	0.0136 (6)	
N4A	0.5456 (4)	0.2375 (3)	1.06297 (16)	0.0154 (6)	
C1A	0.4380 (5)	0.2851 (4)	0.84447 (18)	0.0127 (7)	
C2A	0.5381 (4)	0.1691 (4)	0.88527 (18)	0.0125 (6)	
C3A	0.5721 (4)	0.1543 (4)	0.95672 (18)	0.0126 (7)	
H3A	0.6389	0.0787	0.9827	0.015*	
C4A	0.5064 (4)	0.2520 (4)	0.98843 (18)	0.0128 (7)	
C5A	0.4078 (5)	0.3681 (4)	0.95041 (19)	0.0152 (7)	
H5A	0.3643	0.4335	0.9730	0.018*	
C6A	0.3764 (5)	0.3842 (4)	0.87965 (18)	0.0139 (7)	
H6A	0.3131	0.4620	0.8542	0.017*	
C7A	0.2916 (5)	0.4404 (4)	0.67163 (18)	0.0131 (7)	
C8A	0.3760 (5)	0.3548 (4)	0.62647 (19)	0.0183 (7)	
H8A	0.4529	0.2834	0.6552	0.027*	
H8B	0.4442	0.4068	0.5905	0.027*	
H8C	0.2853	0.3194	0.6048	0.027*	
C9A	0.1823 (5)	0.5658 (4)	0.63623 (18)	0.0138 (7)	
C10A	0.1036 (5)	0.6493 (4)	0.67685 (19)	0.0157 (7)	
H10A	0.1173	0.6256	0.7257	0.019*	
C11A	0.0055 (5)	0.7671 (4)	0.6442 (2)	0.0168 (7)	
C12A	−0.0155 (5)	0.8030 (4)	0.5704 (2)	0.0207 (8)	
H12A	−0.0797	0.8830	0.5483	0.025*	
C13A	0.0596 (6)	0.7191 (4)	0.5309 (2)	0.0227 (8)	
H13A	0.0440	0.7424	0.4821	0.027*	
C14A	0.1580 (5)	0.6005 (4)	0.56287 (19)	0.0189 (8)	
H14A	0.2075	0.5445	0.5357	0.023*	
C15A	−0.0621 (5)	0.8217 (4)	0.7541 (2)	0.0223 (8)	
H15A	−0.1352	0.8862	0.7723	0.033*	
H15B	0.0593	0.8200	0.7686	0.033*	
H15C	−0.1019	0.7358	0.7720	0.033*	
O1B	0.3655 (4)	0.3835 (3)	0.45297 (14)	0.0202 (6)	
O2B	0.4931 (4)	0.1929 (3)	0.51179 (14)	0.0258 (7)	
O3B	0.8328 (4)	−0.0989 (3)	0.39840 (15)	0.0215 (6)	
O4B	0.7842 (4)	−0.0858 (3)	0.28765 (15)	0.0234 (6)	
O5B	0.1203 (4)	0.6180 (3)	−0.00270 (14)	0.0187 (6)	
N1B	0.3221 (4)	0.4301 (3)	0.31624 (16)	0.0150 (6)	
H1NB	0.288 (6)	0.456 (5)	0.3542 (17)	0.022*	
N2B	0.2573 (4)	0.4970 (3)	0.25056 (16)	0.0140 (6)	
N3B	0.4467 (4)	0.2707 (3)	0.45574 (16)	0.0161 (6)	
N4B	0.7603 (4)	−0.0432 (3)	0.34084 (17)	0.0167 (6)	
C1B	0.4252 (4)	0.3144 (4)	0.32377 (18)	0.0124 (7)	
C2B	0.4874 (5)	0.2337 (4)	0.39022 (18)	0.0121 (6)	
C3B	0.5970 (5)	0.1153 (4)	0.39627 (19)	0.0136 (7)	
H3B	0.6383	0.0645	0.4402	0.016*	
C4B	0.6422 (4)	0.0759 (4)	0.33599 (18)	0.0128 (7)	
C5B	0.5822 (5)	0.1513 (4)	0.26873 (19)	0.0145 (7)	
H5B	0.6129	0.1218	0.2285	0.017*	
C6B	0.4787 (5)	0.2677 (4)	0.26357 (18)	0.0131 (7)	
H6B	0.4418	0.3185	0.2191	0.016*	
C7B	0.1651 (4)	0.6103 (4)	0.24548 (18)	0.0129 (7)	
C8B	0.1308 (5)	0.6726 (4)	0.3065 (2)	0.0193 (8)	
H8D	0.1039	0.6061	0.3475	0.029*	
H8E	0.0319	0.7399	0.2952	0.029*	
H8F	0.2345	0.7117	0.3160	0.029*	
C9B	0.0979 (5)	0.6790 (4)	0.17307 (19)	0.0136 (7)	
C10B	0.1345 (5)	0.6197 (4)	0.11704 (19)	0.0144 (7)	
H10B	0.2000	0.5369	0.1250	0.017*	
C11B	0.0731 (5)	0.6842 (4)	0.0492 (2)	0.0147 (7)	
C12B	−0.0255 (5)	0.8084 (4)	0.0359 (2)	0.0170 (7)	
H12B	−0.0668	0.8509	−0.0095	0.020*	
C13B	−0.0604 (5)	0.8667 (4)	0.0919 (2)	0.0183 (7)	
H13B	−0.1250	0.9498	0.0838	0.022*	
C14B	−0.0003 (5)	0.8030 (4)	0.1601 (2)	0.0166 (7)	
H14B	−0.0259	0.8434	0.1971	0.020*	
C15B	0.0306 (6)	0.6672 (4)	−0.0701 (2)	0.0216 (8)	
H15D	0.0630	0.6079	−0.0998	0.032*	
H15E	0.0645	0.7535	−0.0919	0.032*	
H15F	−0.0957	0.6730	−0.0635	0.032*	

### Atomic displacement parameters (Å^2^)

**Table:**

	*U*^11^	*U*^22^	*U*^33^	*U*^12^	*U*^13^	*U*^23^
O1A	0.0298 (14)	0.0200 (14)	0.0094 (12)	0.0053 (11)	−0.0047 (10)	−0.0060 (10)
O2A	0.0204 (13)	0.0123 (13)	0.0192 (13)	0.0049 (10)	−0.0053 (10)	−0.0018 (10)
O3A	0.0217 (13)	0.0195 (14)	0.0148 (12)	0.0016 (11)	−0.0053 (10)	−0.0002 (11)
O4A	0.0352 (16)	0.0217 (15)	0.0124 (12)	0.0048 (12)	−0.0008 (11)	−0.0064 (11)
O5A	0.0258 (14)	0.0187 (15)	0.0221 (14)	0.0074 (11)	−0.0007 (11)	−0.0040 (12)
N1A	0.0162 (14)	0.0105 (14)	0.0102 (14)	0.0036 (11)	−0.0039 (11)	−0.0015 (11)
N2A	0.0177 (14)	0.0137 (15)	0.0113 (14)	0.0022 (12)	−0.0046 (11)	−0.0023 (12)
N3A	0.0145 (14)	0.0131 (15)	0.0129 (14)	0.0011 (11)	−0.0005 (11)	−0.0038 (12)
N4A	0.0208 (15)	0.0144 (15)	0.0106 (14)	−0.0020 (12)	0.0000 (11)	−0.0021 (12)
C1A	0.0143 (15)	0.0129 (17)	0.0114 (16)	−0.0030 (13)	0.0018 (12)	−0.0031 (13)
C2A	0.0142 (15)	0.0134 (17)	0.0103 (15)	0.0000 (12)	0.0020 (12)	−0.0045 (13)
C3A	0.0116 (15)	0.0131 (17)	0.0123 (16)	−0.0022 (12)	0.0000 (12)	−0.0012 (13)
C4A	0.0129 (15)	0.0132 (17)	0.0106 (15)	−0.0007 (13)	−0.0003 (12)	0.0001 (13)
C5A	0.0189 (17)	0.0141 (18)	0.0124 (16)	−0.0002 (13)	0.0016 (13)	−0.0036 (13)
C6A	0.0174 (16)	0.0116 (17)	0.0114 (16)	0.0028 (13)	−0.0007 (12)	−0.0020 (13)
C7A	0.0159 (16)	0.0121 (17)	0.0110 (15)	−0.0006 (13)	−0.0008 (12)	−0.0026 (13)
C8A	0.0268 (19)	0.0173 (19)	0.0099 (16)	0.0026 (15)	−0.0002 (13)	−0.0037 (14)
C9A	0.0156 (16)	0.0128 (17)	0.0111 (16)	−0.0013 (13)	−0.0036 (12)	0.0005 (13)
C10A	0.0178 (16)	0.0157 (18)	0.0129 (16)	−0.0044 (14)	−0.0027 (13)	−0.0011 (14)
C11A	0.0150 (16)	0.0164 (18)	0.0191 (18)	−0.0014 (14)	0.0014 (13)	−0.0047 (15)
C12A	0.0229 (19)	0.0181 (19)	0.0172 (18)	0.0029 (15)	−0.0048 (14)	0.0015 (15)
C13A	0.029 (2)	0.025 (2)	0.0107 (17)	−0.0002 (16)	−0.0047 (14)	0.0008 (15)
C14A	0.0234 (18)	0.021 (2)	0.0103 (16)	0.0003 (15)	−0.0016 (13)	−0.0021 (14)
C15A	0.0220 (18)	0.024 (2)	0.0209 (19)	0.0018 (16)	0.0050 (15)	−0.0076 (16)
O1B	0.0289 (14)	0.0166 (14)	0.0147 (13)	0.0064 (11)	0.0003 (10)	−0.0066 (11)
O2B	0.0438 (18)	0.0211 (15)	0.0091 (12)	0.0063 (13)	0.0032 (11)	−0.0009 (11)
O3B	0.0248 (14)	0.0194 (14)	0.0169 (13)	0.0063 (11)	−0.0050 (11)	−0.0012 (11)
O4B	0.0310 (15)	0.0211 (15)	0.0178 (14)	0.0088 (12)	−0.0018 (11)	−0.0089 (12)
O5B	0.0233 (13)	0.0187 (14)	0.0136 (13)	0.0026 (11)	−0.0026 (10)	−0.0051 (11)
N1B	0.0193 (15)	0.0147 (15)	0.0098 (14)	0.0031 (12)	−0.0009 (11)	−0.0029 (12)
N2B	0.0140 (13)	0.0140 (15)	0.0118 (14)	0.0018 (11)	0.0000 (11)	−0.0001 (12)
N3B	0.0212 (15)	0.0158 (16)	0.0106 (14)	0.0002 (12)	0.0018 (11)	−0.0026 (12)
N4B	0.0182 (14)	0.0141 (16)	0.0171 (15)	0.0020 (12)	0.0006 (11)	−0.0040 (12)
C1B	0.0127 (15)	0.0120 (17)	0.0126 (16)	−0.0002 (12)	0.0004 (12)	−0.0034 (13)
C2B	0.0152 (15)	0.0137 (17)	0.0075 (15)	−0.0005 (13)	0.0015 (12)	−0.0029 (13)
C3B	0.0153 (16)	0.0132 (17)	0.0110 (15)	−0.0008 (13)	−0.0004 (12)	−0.0007 (13)
C4B	0.0132 (15)	0.0102 (16)	0.0136 (16)	0.0026 (12)	−0.0008 (12)	−0.0019 (13)
C5B	0.0146 (16)	0.0168 (18)	0.0130 (16)	−0.0019 (13)	−0.0016 (12)	−0.0048 (14)
C6B	0.0165 (16)	0.0121 (17)	0.0088 (15)	0.0003 (13)	−0.0015 (12)	0.0005 (13)
C7B	0.0114 (14)	0.0128 (17)	0.0137 (16)	0.0007 (12)	0.0006 (12)	−0.0024 (13)
C8B	0.0223 (18)	0.0192 (19)	0.0165 (17)	0.0050 (15)	−0.0016 (14)	−0.0075 (15)
C9B	0.0122 (15)	0.0140 (17)	0.0134 (16)	−0.0010 (13)	0.0002 (12)	−0.0015 (13)
C10B	0.0131 (15)	0.0128 (17)	0.0160 (17)	0.0012 (13)	0.0000 (12)	−0.0019 (14)
C11B	0.0137 (15)	0.0126 (17)	0.0181 (17)	−0.0028 (13)	0.0001 (13)	−0.0035 (14)
C12B	0.0149 (16)	0.0154 (18)	0.0193 (18)	0.0004 (13)	−0.0034 (13)	−0.0019 (14)
C13B	0.0192 (17)	0.0120 (17)	0.0211 (19)	0.0021 (14)	0.0010 (14)	−0.0006 (14)
C14B	0.0169 (16)	0.0155 (18)	0.0160 (17)	0.0005 (13)	0.0025 (13)	−0.0022 (14)
C15B	0.026 (2)	0.024 (2)	0.0146 (17)	−0.0027 (16)	−0.0031 (14)	−0.0044 (15)

### Geometric parameters (Å, º)

**Table:**

O1A—N3A	1.243 (4)	O1B—N3B	1.261 (4)
O2A—N3A	1.229 (4)	O2B—N3B	1.224 (4)
O3A—N4A	1.232 (4)	O3B—N4B	1.236 (4)
O4A—N4A	1.228 (4)	O4B—N4B	1.230 (4)
O5A—C11A	1.367 (5)	O5B—C11B	1.383 (5)
O5A—C15A	1.427 (5)	O5B—C15B	1.438 (5)
N1A—C1A	1.353 (4)	N1B—C1B	1.347 (5)
N1A—N2A	1.374 (4)	N1B—N2B	1.370 (4)
N1A—H1NA	0.868 (19)	N1B—H1NB	0.873 (19)
N2A—C7A	1.294 (5)	N2B—C7B	1.293 (5)
N3A—C2A	1.447 (5)	N3B—C2B	1.444 (4)
N4A—C4A	1.456 (4)	N4B—C4B	1.439 (5)
C1A—C6A	1.414 (5)	C1B—C6B	1.418 (5)
C1A—C2A	1.425 (5)	C1B—C2B	1.419 (5)
C2A—C3A	1.388 (5)	C2B—C3B	1.397 (5)
C3A—C4A	1.367 (5)	C3B—C4B	1.367 (5)
C3A—H3A	0.9300	C3B—H3B	0.9300
C4A—C5A	1.402 (5)	C4B—C5B	1.409 (5)
C5A—C6A	1.370 (5)	C5B—C6B	1.360 (5)
C5A—H5A	0.9300	C5B—H5B	0.9300
C6A—H6A	0.9300	C6B—H6B	0.9300
C7A—C9A	1.492 (5)	C7B—C9B	1.489 (5)
C7A—C8A	1.496 (5)	C7B—C8B	1.498 (5)
C8A—H8A	0.9600	C8B—H8D	0.9600
C8A—H8B	0.9600	C8B—H8E	0.9600
C8A—H8C	0.9600	C8B—H8F	0.9600
C9A—C14A	1.398 (5)	C9B—C14B	1.393 (5)
C9A—C10A	1.401 (5)	C9B—C10B	1.394 (5)
C10A—C11A	1.382 (5)	C10B—C11B	1.393 (5)
C10A—H10A	0.9300	C10B—H10B	0.9300
C11A—C12A	1.402 (5)	C11B—C12B	1.395 (5)
C12A—C13A	1.380 (6)	C12B—C13B	1.384 (6)
C12A—H12A	0.9300	C12B—H12B	0.9300
C13A—C14A	1.386 (6)	C13B—C14B	1.394 (5)
C13A—H13A	0.9300	C13B—H13B	0.9300
C14A—H14A	0.9300	C14B—H14B	0.9300
C15A—H15A	0.9600	C15B—H15D	0.9600
C15A—H15B	0.9600	C15B—H15E	0.9600
C15A—H15C	0.9600	C15B—H15F	0.9600
			
C11A—O5A—C15A	117.4 (3)	C11B—O5B—C15B	116.8 (3)
C1A—N1A—N2A	118.3 (3)	C1B—N1B—N2B	119.4 (3)
C1A—N1A—H1NA	117 (3)	C1B—N1B—H1NB	118 (3)
N2A—N1A—H1NA	125 (3)	N2B—N1B—H1NB	122 (3)
C7A—N2A—N1A	117.6 (3)	C7B—N2B—N1B	117.2 (3)
O2A—N3A—O1A	122.0 (3)	O2B—N3B—O1B	122.2 (3)
O2A—N3A—C2A	119.2 (3)	O2B—N3B—C2B	119.4 (3)
O1A—N3A—C2A	118.7 (3)	O1B—N3B—C2B	118.4 (3)
O4A—N4A—O3A	123.7 (3)	O4B—N4B—O3B	122.7 (3)
O4A—N4A—C4A	117.9 (3)	O4B—N4B—C4B	118.6 (3)
O3A—N4A—C4A	118.3 (3)	O3B—N4B—C4B	118.6 (3)
N1A—C1A—C6A	120.4 (3)	N1B—C1B—C6B	120.2 (3)
N1A—C1A—C2A	122.4 (3)	N1B—C1B—C2B	123.3 (3)
C6A—C1A—C2A	117.2 (3)	C6B—C1B—C2B	116.4 (3)
C3A—C2A—C1A	121.1 (3)	C3B—C2B—C1B	122.0 (3)
C3A—C2A—N3A	116.2 (3)	C3B—C2B—N3B	115.6 (3)
C1A—C2A—N3A	122.7 (3)	C1B—C2B—N3B	122.3 (3)
C4A—C3A—C2A	119.3 (3)	C4B—C3B—C2B	118.3 (3)
C4A—C3A—H3A	120.3	C4B—C3B—H3B	120.8
C2A—C3A—H3A	120.3	C2B—C3B—H3B	120.8
C3A—C4A—C5A	121.7 (3)	C3B—C4B—C5B	122.0 (3)
C3A—C4A—N4A	119.2 (3)	C3B—C4B—N4B	119.2 (3)
C5A—C4A—N4A	119.1 (3)	C5B—C4B—N4B	118.8 (3)
C6A—C5A—C4A	119.2 (3)	C6B—C5B—C4B	119.0 (3)
C6A—C5A—H5A	120.4	C6B—C5B—H5B	120.5
C4A—C5A—H5A	120.4	C4B—C5B—H5B	120.5
C5A—C6A—C1A	121.4 (3)	C5B—C6B—C1B	122.2 (3)
C5A—C6A—H6A	119.3	C5B—C6B—H6B	118.9
C1A—C6A—H6A	119.3	C1B—C6B—H6B	118.9
N2A—C7A—C9A	115.5 (3)	N2B—C7B—C9B	115.1 (3)
N2A—C7A—C8A	126.2 (3)	N2B—C7B—C8B	123.7 (3)
C9A—C7A—C8A	118.2 (3)	C9B—C7B—C8B	121.2 (3)
C7A—C8A—H8A	109.5	C7B—C8B—H8D	109.5
C7A—C8A—H8B	109.5	C7B—C8B—H8E	109.5
H8A—C8A—H8B	109.5	H8D—C8B—H8E	109.5
C7A—C8A—H8C	109.5	C7B—C8B—H8F	109.5
H8A—C8A—H8C	109.5	H8D—C8B—H8F	109.5
H8B—C8A—H8C	109.5	H8E—C8B—H8F	109.5
C14A—C9A—C10A	119.8 (3)	C14B—C9B—C10B	119.1 (3)
C14A—C9A—C7A	120.5 (3)	C14B—C9B—C7B	120.9 (3)
C10A—C9A—C7A	119.7 (3)	C10B—C9B—C7B	119.9 (3)
C11A—C10A—C9A	119.9 (3)	C11B—C10B—C9B	120.1 (3)
C11A—C10A—H10A	120.0	C11B—C10B—H10B	120.0
C9A—C10A—H10A	120.0	C9B—C10B—H10B	120.0
O5A—C11A—C10A	124.5 (3)	O5B—C11B—C10B	115.3 (3)
O5A—C11A—C12A	115.4 (3)	O5B—C11B—C12B	123.6 (3)
C10A—C11A—C12A	120.2 (4)	C10B—C11B—C12B	121.0 (3)
C13A—C12A—C11A	119.6 (4)	C13B—C12B—C11B	118.4 (3)
C13A—C12A—H12A	120.2	C13B—C12B—H12B	120.8
C11A—C12A—H12A	120.2	C11B—C12B—H12B	120.8
C12A—C13A—C14A	121.0 (4)	C12B—C13B—C14B	121.1 (4)
C12A—C13A—H13A	119.5	C12B—C13B—H13B	119.4
C14A—C13A—H13A	119.5	C14B—C13B—H13B	119.4
C13A—C14A—C9A	119.5 (4)	C9B—C14B—C13B	120.2 (4)
C13A—C14A—H14A	120.2	C9B—C14B—H14B	119.9
C9A—C14A—H14A	120.2	C13B—C14B—H14B	119.9
O5A—C15A—H15A	109.5	O5B—C15B—H15D	109.5
O5A—C15A—H15B	109.5	O5B—C15B—H15E	109.5
H15A—C15A—H15B	109.5	H15D—C15B—H15E	109.5
O5A—C15A—H15C	109.5	O5B—C15B—H15F	109.5
H15A—C15A—H15C	109.5	H15D—C15B—H15F	109.5
H15B—C15A—H15C	109.5	H15E—C15B—H15F	109.5
			
C1A—N1A—N2A—C7A	172.4 (3)	C1B—N1B—N2B—C7B	−177.2 (3)
N2A—N1A—C1A—C6A	−1.9 (5)	N2B—N1B—C1B—C6B	5.4 (5)
N2A—N1A—C1A—C2A	179.5 (3)	N2B—N1B—C1B—C2B	−175.2 (3)
N1A—C1A—C2A—C3A	179.2 (3)	N1B—C1B—C2B—C3B	−178.8 (3)
C6A—C1A—C2A—C3A	0.5 (5)	C6B—C1B—C2B—C3B	0.7 (5)
N1A—C1A—C2A—N3A	2.5 (5)	N1B—C1B—C2B—N3B	−0.9 (5)
C6A—C1A—C2A—N3A	−176.2 (3)	C6B—C1B—C2B—N3B	178.5 (3)
O2A—N3A—C2A—C3A	2.4 (5)	O2B—N3B—C2B—C3B	−7.3 (5)
O1A—N3A—C2A—C3A	−176.6 (3)	O1B—N3B—C2B—C3B	172.1 (3)
O2A—N3A—C2A—C1A	179.2 (3)	O2B—N3B—C2B—C1B	174.7 (3)
O1A—N3A—C2A—C1A	0.2 (5)	O1B—N3B—C2B—C1B	−5.9 (5)
C1A—C2A—C3A—C4A	0.8 (5)	C1B—C2B—C3B—C4B	−1.2 (5)
N3A—C2A—C3A—C4A	177.7 (3)	N3B—C2B—C3B—C4B	−179.1 (3)
C2A—C3A—C4A—C5A	−1.1 (5)	C2B—C3B—C4B—C5B	0.3 (5)
C2A—C3A—C4A—N4A	−178.7 (3)	C2B—C3B—C4B—N4B	177.7 (3)
O4A—N4A—C4A—C3A	−177.2 (3)	O4B—N4B—C4B—C3B	171.9 (3)
O3A—N4A—C4A—C3A	3.2 (5)	O3B—N4B—C4B—C3B	−7.7 (5)
O4A—N4A—C4A—C5A	5.2 (5)	O4B—N4B—C4B—C5B	−10.6 (5)
O3A—N4A—C4A—C5A	−174.4 (3)	O3B—N4B—C4B—C5B	169.8 (3)
C3A—C4A—C5A—C6A	0.0 (5)	C3B—C4B—C5B—C6B	1.1 (5)
N4A—C4A—C5A—C6A	177.6 (3)	N4B—C4B—C5B—C6B	−176.3 (3)
C4A—C5A—C6A—C1A	1.3 (5)	C4B—C5B—C6B—C1B	−1.7 (5)
N1A—C1A—C6A—C5A	179.7 (3)	N1B—C1B—C6B—C5B	−179.8 (3)
C2A—C1A—C6A—C5A	−1.5 (5)	C2B—C1B—C6B—C5B	0.8 (5)
N1A—N2A—C7A—C9A	179.8 (3)	N1B—N2B—C7B—C9B	179.9 (3)
N1A—N2A—C7A—C8A	−1.3 (5)	N1B—N2B—C7B—C8B	1.7 (5)
N2A—C7A—C9A—C14A	180.0 (3)	N2B—C7B—C9B—C14B	−178.8 (3)
C8A—C7A—C9A—C14A	1.0 (5)	C8B—C7B—C9B—C14B	−0.5 (5)
N2A—C7A—C9A—C10A	0.1 (5)	N2B—C7B—C9B—C10B	0.6 (5)
C8A—C7A—C9A—C10A	−179.0 (3)	C8B—C7B—C9B—C10B	178.9 (3)
C14A—C9A—C10A—C11A	−1.4 (5)	C14B—C9B—C10B—C11B	0.0 (5)
C7A—C9A—C10A—C11A	178.5 (3)	C7B—C9B—C10B—C11B	−179.4 (3)
C15A—O5A—C11A—C10A	−2.6 (5)	C15B—O5B—C11B—C10B	167.7 (3)
C15A—O5A—C11A—C12A	178.0 (3)	C15B—O5B—C11B—C12B	−13.8 (5)
C9A—C10A—C11A—O5A	−179.3 (3)	C9B—C10B—C11B—O5B	178.6 (3)
C9A—C10A—C11A—C12A	0.1 (5)	C9B—C10B—C11B—C12B	0.0 (5)
O5A—C11A—C12A—C13A	−179.4 (3)	O5B—C11B—C12B—C13B	−178.2 (3)
C10A—C11A—C12A—C13A	1.1 (6)	C10B—C11B—C12B—C13B	0.3 (5)
C11A—C12A—C13A—C14A	−1.0 (6)	C11B—C12B—C13B—C14B	−0.6 (5)
C12A—C13A—C14A—C9A	−0.3 (6)	C10B—C9B—C14B—C13B	−0.2 (5)
C10A—C9A—C14A—C13A	1.5 (6)	C7B—C9B—C14B—C13B	179.2 (3)
C7A—C9A—C14A—C13A	−178.4 (3)	C12B—C13B—C14B—C9B	0.5 (6)

### Hydrogen-bond geometry (Å, º)

**Table:**

*D*—H···*A*	*D*—H	H···*A*	*D*···*A*	*D*—H···*A*
N1*A*—H1*NA*···O1*A*	0.87 (3)	1.94 (4)	2.611 (4)	133 (3)
N1*B*—H1*NB*···O1*B*	0.88 (4)	1.96 (3)	2.611 (4)	130 (4)
C5*B*—H5*B*···O3*A*^i^	0.93	2.59	3.462 (5)	156
C6*B*—H6*B*···O4*A*^i^	0.93	2.50	3.277 (4)	141
C8*A*—H8*C*···O3*B*^ii^	0.96	2.57	3.376 (5)	142
C8*B*—H8*E*···O4*B*^iii^	0.96	2.45	3.402 (5)	170
C12*B*—H12*B*···O2*A*^iv^	0.93	2.57	3.447 (5)	157
C13*A*—H13*A*···O3*B*^iii^	0.93	2.48	3.204 (5)	135
C13*B*—H13*B*···O3*A*^iv^	0.93	2.55	3.448 (5)	162
C14*B*—H14*B*···O4*B*^iii^	0.93	2.47	3.338 (5)	155
C15*B*—H15*E*···O3*A*^v^	0.96	2.57	3.301 (5)	133

Symmetry codes: (i) *x*, *y*, *z*−1; (ii) −*x*+1, −*y*, −*z*+1; (iii) *x*−1, *y*+1, *z*; (iv) *x*−1, *y*+1, *z*−1; (v) −*x*+1, −*y*+1, −*z*+1.
